# Transient heating of Pd nanoparticles studied by x-ray diffraction with time of arrival photon detection

**DOI:** 10.1063/5.0189052

**Published:** 2024-07-03

**Authors:** Simon Chung, Vedran Vonk, David Pennicard, Heinz Graafsma, Andreas Stierle

**Affiliations:** 1 Centre for X-ray and Nano Science CXNS, Deutsches Elektronen-Synchrotron DESY, Notkestr. 85, 22607 Hamburg, Germany; 2 Department of Electrical and Computer Engineering, Rice University, Houston, Texas 77005, USA; 3 Deutsches Elektronen-Synchrotron DESY, Notkestr. 85, 22607 Hamburg, Germany; 4 Mid-Sweden University, Sundsvall, Sweden; 5 Fachbereich Physik, Universität Hamburg, Jungiusstrasse 11, 20355 Hamburg, Germany

## Abstract

Pulsed laser heating of an ensemble of Pd nanoparticles, supported by a MgO substrate, is studied by x-ray diffraction. By time-resolved Bragg peak shift measurements due to thermal lattice expansion, the transient temperature of the Pd nanoparticles is determined, which quickly rises by at least 100 K upon laser excitation and then decays within 90 ns. The diffraction experiments were carried out using a Cu x-ray tube, giving continuous radiation, and the hybrid pixel detector Timepix3 operating with single photon counting in a time-of-arrival mode. This type of detection scheme does not require time-consuming scanning of the pump-probe delay. The experimental time resolution is estimated at 15 ± 5 ns, which is very close to the detector's limit and matches with the 7 ns laser pulse duration. Compared to bulk metal single crystals, it is discussed that the maximum temperature reached by the Pd nanoparticles is higher and their cooling rate is lower. These effects are explained by the oxide support having a lower heat conductivity.

Heat transport in thin films, nanoparticles, and surfaces plays an important role in applications such as electronics and heterogeneous catalysis. In the former case, the control over the heat dissipation is crucial for the development of ever smaller transistors, the latter process is poorly understood in relation to heat transport due to exo- or endothermic reactions, which must have a significant impact on the reaction rates. Heat transport phenomena find their basis in the elastic properties, which in small objects differ significantly from the bulk and are governed by surface effects. Advances in nanomaterials synthesis have enabled the so-called phonon engineering,^1^ which aims at optimizing the thermal conductivity independently from other material properties.^2,3^ These developments call for experimental access to thermal properties on the nanoscale, such as temperature, heat conduction coefficients, and elastic responses. In the case of the study of supported nanoparticles, using a laser pump-x-ray probe scheme allows the probe of inter-atomic distances and with that addresses directly the elastic properties of the material.^4^

Here, we present experiments on the transient temperature and heat transfer of Pd nanoparticles supported by a MgO(001) substrate. This is a model system for a heterogeneous catalyst used in exhaust cleaning. During CO oxidation, Pd nanoparticles may change their shape by the formation of a thin oxide layer on their facets.^5,6^ Such effect raises questions concerning the catalyst's active phase, the emergence of possible new active sites, and relevant time-scales under working conditions when dynamic reaction fronts are expected. The experiment described here consists of heating the sample with a pulsed nanosecond-laser and measuring the corresponding response of the Pd-111 Bragg reflection. The experimental time resolution lies in the 10–20 ns range, which means that the “thermal tail” of the heat dissipation is probed.

Laser-pump x-ray probe experiments are usually performed stroboscopically with pulsed beams and by scanning the delay time between them. For example, the electronic gating capabilities of such a detector enable pump-probe experiments with a time resolution of around 100 ns.^7^ The latest generation of two-dimensional (2D) hybrid pixel detectors, the so-called Timepix and described hereafter, allows for designing experiments without the need for varying the delay time between the pump and probe pulses utilizing pixel detectors with electronic gating. The Timepix detector achieves time-resolution by binning the photon detection events onto a time axis relative to a clock, similar to stroboscopic measurement schemes with single-element detectors developed in the 1990s.^8^ The Timepix implements this in a pixel detector with small pixels enabling measurements of small shifts in the peak position, which was not possible with single-element detectors where an angular axis will need to be scanned. The Timepix detector combines the benefits of the two approaches for time-resolved experiments, point detectors with time stamping, and pixel detectors with electronic gating. Utilizing a Timepix3 detector, all the diffracted photons are collected continuously in between the laser pulses, and the sampled delay times are determined from a binning procedure during the data analysis. This speeds up the data collection time linearly with a factor given by the number of delay times sampled in conventional pump-probe experiments. Especially for x-ray lab sources with relatively low flux, this means that the total data collection time can be shortened from weeks to days.

The Timepix3 is a hybrid pixel detector readout chip developed by the Medipix3 collaboration.^9,10^ The 256 × 256 pixel array consisting of $55\times 55\;\mu m^{2}$ sized pixels can detect individual x-ray photons striking each pixel, by amplifying the signal pulse produced by each photon hit and comparing this to a user defined threshold. The detector is able to process events up to a hit rate of 40 Mhits/cm^2^/s, making it possible perform experiments with moderate x-ray flux. Each time a photon hits a pixel, a data packet is recorded, containing the X, Y coordinates of the pixel and a timestamp with 1.6 ns time binning with a 640 MHz clock [“time of arrival” (ToA)].^11^ Additionally, by measuring the time period that the signal pulse is above threshold [“time over threshold” (ToT)], Timepix3 also provides an approximate measurement of each photon's energy.

The experiments were performed at the DESY NanoLab.^12^ Figure 1(a) shows a schematic of the time-resolved x-ray diffraction experiment. The Pd sample was deposited by molecular beam epitaxy on a MgO(001) substrate in an ultra-high vacuum system, with a base pressure less than $1\times 10^{-10}$ mbar. The MgO(001) substrate was prepared by cleaning in an ultrasonic bath of isopropanol and ethanol for 15 min each, then annealing in a tube furnace in air at 1273 K for 1 h prior to introducing it to the vacuum system. The substrate was degassed for 1 h at 770 K, followed by two cycles of Ar ion sputtering ( $7\times 10^{-6}$ mbar at 1 and 0.6 keV for the second cycle) for 1 h and annealing in oxygen. Directly following the surface preparation, palladium was deposited for 30 min at a rate of 4.63 Å/min at 500 K. Figure 1(a) shows the measured x-ray reflection curve of the sample. An electron density profile was fitted to the data using the Parratt formalism.^13^ The height of the Pd nanoparticles were determined to be 13.9 nm with a coverage of approximately 80% [see Fig. 2(a)].

**FIG. 1.**
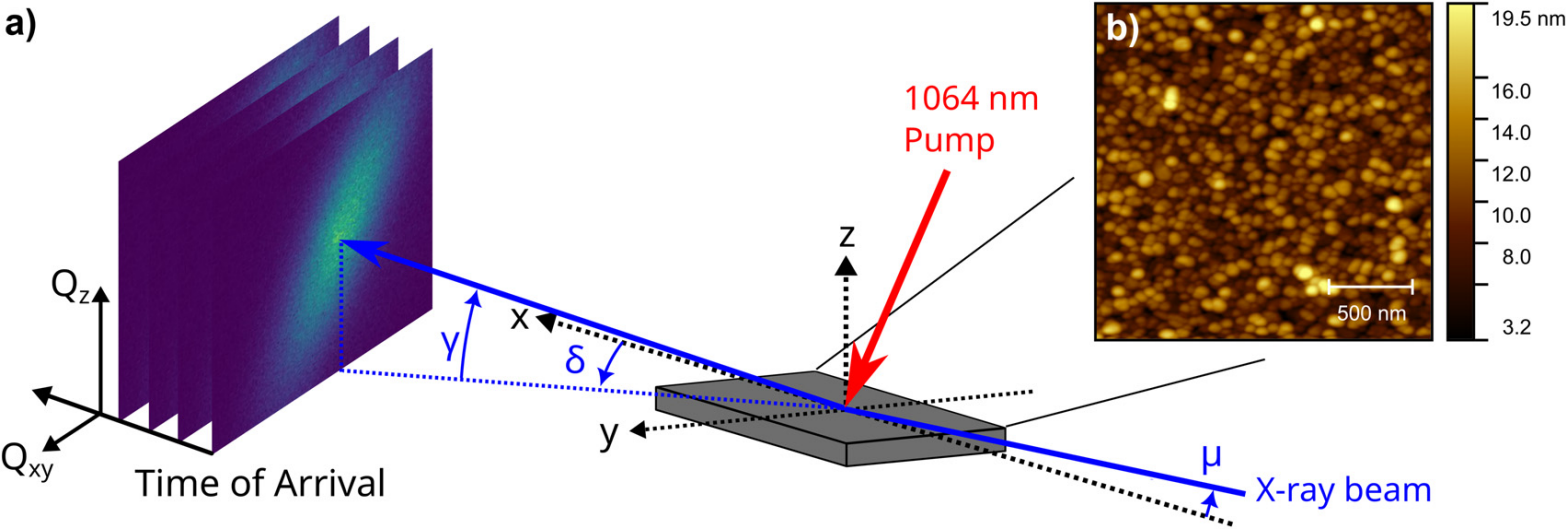
(a) Schematic of the experiment showing the diffraction geometry and laser pump beam. The angles are defined as *δ* and *γ*, which are the detector in-plane and out-of-plane angles with respect to the sample surface normal, z, while *μ* is the x-ray incident angle along the xz sample plane. The diffracted peaks are recorded by binning the data stream of the Timepix3 detector into separate 2D images. (b) Atomic force microscopy image of the Pd nanoparticle sample.

Although the coverage of Pd metal is relatively high, atomic force microscopy does show the appearance of isolated particles with lateral sizes in the 50–100 nm range [see Fig. 1(b)]. Since the morphology does not form a closed film, it is expected that the particles will respond isotropically to a heat impulse and will not be laterally constrained during thermal expansion.

**FIG. 2.**
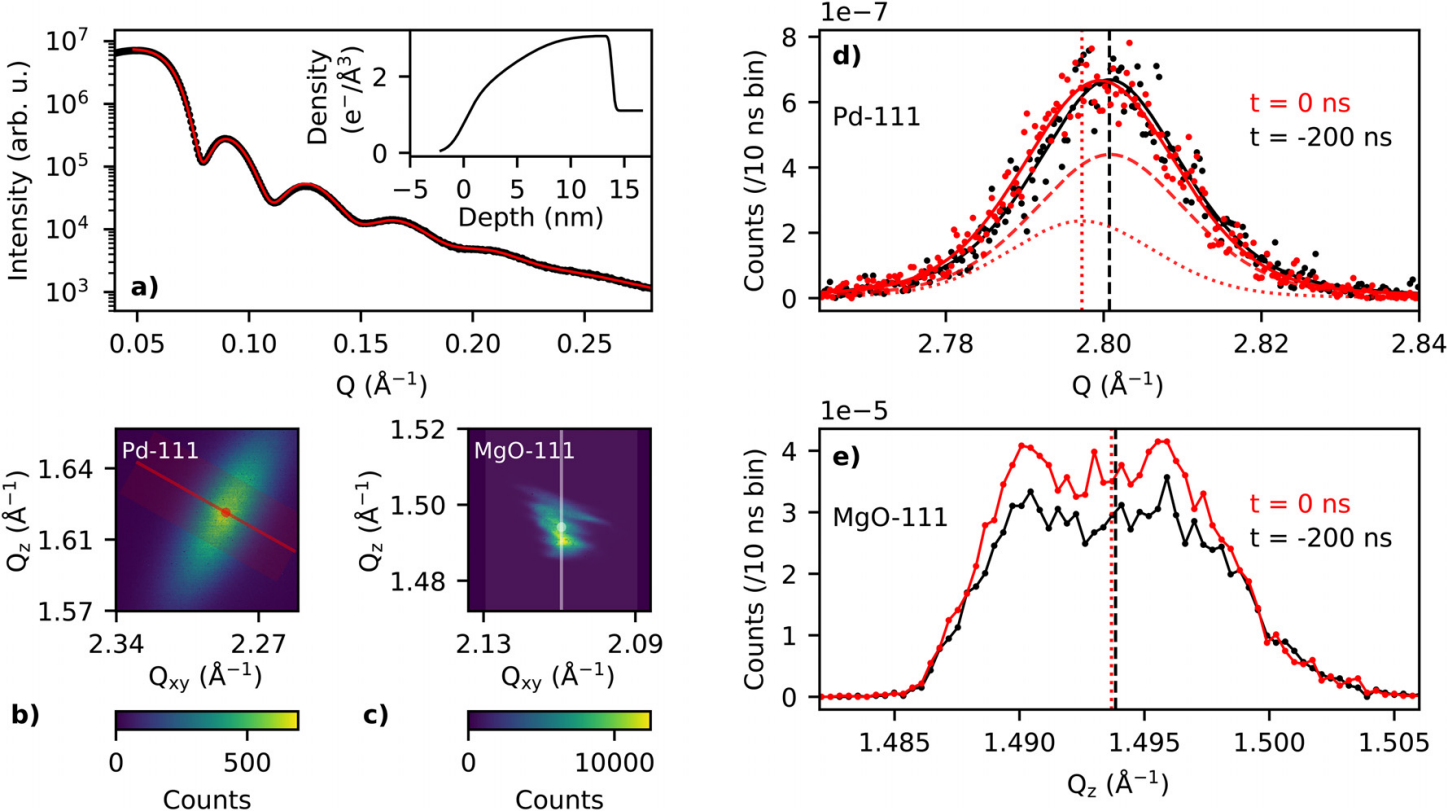
(a) x-ray reflectivity (data, black) along with fit (red) and fitted density in the inset. (b) Detector image of Pd-111 reflection of events in the 11 *μ*s ToA range; red line corresponds to profile in (d). (c) Detector image of MgO-111 reflection of events in the 11 *μ*s ToA range; white line corresponds to profile in (e). (d) Line profile of the Pd-111 reflection, across the radial direction, at t = 0 and −200 ns; vertical lines indicate the center of pseudo-Voigt fits. Black lines show one-component fit before time zero and red lines show two-component fit, representing the pumped (dotted line) and unpumped (dashed line) portions of the sample. (e) Line profile of the MgO-111 reflection, across the perpendicular direction, at t = 0 and −200 ns; vertical lines indicate the center of mass of the peak.

The time-resolved x-ray diffraction experiments were performed in air, and at room temperature, the sample was excited by a 1064 nm Nd:YAG Q-switched laser. The laser delivers 7 ns pulses, and the experiment was performed with a repetition rate of 1 kHz with a pulse energy of 1 mJ. The Cu K*α* x-ray beam was focused into a $0.25\times 0.25\;{\text{mm}}^{2}$ spot, delivering approximately $1\times 10^{7}$ photons/s. The python library PymePix was used for the control and data acquisition for the Timepix3 detector.^14^ An external trigger from a digital delay generator was used as a time zero reference for the Timepix's ToA timestamps and for synchronization with the pump laser. The sample was mounted on a six-circle diffractometer, allowing for diffraction in grazing incidence geometry.^15^ The Timepix3 detector was mounted on the detector arm such that the pixel rows and columns are parallel to the angles *δ* and *γ*, respectively (see Fig. 1). The fixed x-ray angle of incidence *μ* was 5°, resulting in a beam footprint of $2.9\times 0.25\;{\text{mm}}^{2}$. The laser beam, mounted in near-to-normal incidence, had a footprint of approximately 0.8 mm in diameter and was centered at the x-ray beam footprint. The diffraction signal with the laser incident on the sample was allowed to reach a steady state condition prior to acquisition of the time-resolved data.

Data of the Pd-111 reflection were acquired over 20 h, resulting in $9\times 10^{8}$ events and MgO-111 for 100 min consisting of $7\times 10^{8}$ events recorded. Corrections were applied to the data from Timepix3. First, when a photon is absorbed close to the boundary between pixels, its signal can be shared between them, which can result in more than 1 pixel being triggered. To ensure that each photon hit is counted only once, the ToT measurement, which is proportional to the measured energy, was used to exclude hits where the deposited energy was less than half the photon energy.

In cases where a photon is shared between 2 pixels, this will exclude the lower energy hit while accepting the higher-energy one. Additionally, events with abnormally high energy were excluded, due to background radiation such as cosmic rays. Approximately 18% of the recorded hits were excluded due to their ToT value. Second, when a photon hits a pixel, there will be a short time delay before the signal level reaches the threshold level and gets detected. This results in an error of the measured time, called “timewalk,” which varies with the energy deposited in the pixel and was corrected for.^16,17^ Past measurements with synchrotron x-rays in the range of 10–20 keV have demonstrated a time resolution around 19 ns full width at half maximum (FWHM).^18^

The corrected photon detection events were binned according to their pixel position on the detector and into 10 ns temporal windows and normalized for each laser pulse. Figure 2 shows the Pd-111 and MgO-111 reflections resolved according to their and perpendicular, *Q_z_*, direction with respect to the surface and accumulated from −1 to +10 *μ*s with respect to time zero, *t*_0_. The momentum transfer, *Q,* and its in-plane, *Q_xy_*, and out-of-plane, *Q_z_*, components were calculated from the diffraction angles; *δ*, *γ*, and *μ*, illustrated in Fig. 1(a), utilizing the methods described by Lohmeier and Vlieg.^15^

Figure 2 shows line profiles across the Pd-111 in the radial *Q*-direction and the MgO-111 in the *Q_z_*-direction for 10 ns time bins at *t* = −200 ns and *t*_0_. Time zero (*t*_0_) here is defined as the time at the maximum response of the Pd signal, which was also used for the MgO-111 data. These line profiles were obtained by integrating the pixels perpendicular to the desired angular direction, as indicated by the shaded regions in Fig. 2.

The x-rays probe both pumped and unpumped regions of the sample; approximately a third of the x-ray footprint has an overlap with the smaller laser spot. The resulting Bragg peak in such a case consists of the superposition of two components, one of which shifts with laser irradiation and the other remains steady. Figure 2(d) shows this effect; while the high angle flanks of the pumped and unpumped Bragg peaks nearly overlap, the low angle flanks clearly shift. This is indeed an indication that Bragg peak consists of two components, one of which remains at its position and the other shifts due to the laser heating. For this reason, a superposition of two pseudo-Voigt peaks was fitted to the intensity line profile of the Pd-111 reflection for each time bin. The values for the steady, unpumped component were derived and fixed to those obtained from analysis of the peak before laser excitation at time zero. The relative contributions of the pumped and unpumped components were fixed to 1/3 and 2/3 of the total peak area, respectively. The fitted parameters per time bin thus consisted of the total peak area, the position of the pumped component, and a linear background. It was not possible to fit a single analytical peak to the MgO-111 data, due to the inhomogeneous intensity distribution, and therefore, the center of mass was used.

The temporal response of the Pd-111 reflection's momentum transfer, *Q*, in the radial direction is shown in Fig. 3. A decrease in *Q* is observed, indicating an increase in the lattice parameter upon laser excitation. The change in temperature, ΔT, plotted on the right axis, is calculated from the linear thermal expansion coefficient of the bulk (see Table II in the Appendix). The nanoparticle size of the sample used here is large enough to be considered having bulk-like properties, thereby possibly introducing an approximately 5% error.^19^ The temporal response was fitted with an exponentially modified Gaussian,^20^ which is defined as

$$g(t)=\frac{A}{2\tau }\text{exp}[\frac{1}{\tau }(t_{0}-t+\frac{\sigma^{2}}{2\tau })]\text{erfc}\left(\frac{t_{0}+\sigma^{2}/\tau -t}{\sqrt{2}\sigma }\right)+g_{0},$$ (1)

where *g* is the response of the measured quantity, *A* is an amplitude, *t*_0_ is the time at which the sample is excited, *τ* is the exponential decay time of the heat dissipation, *σ* is a temporal broadening parameter, and *g*_0_ is the undisturbed baseline value. This response function can be seen as the result of convolving a shifted exponential with a Gaussian function and its use here is based on semi-empirical grounds. Here, the 7 ns laser pulse heats up the sample after which the heat dissipates exponentially. Therefore, the time-constant *τ* is related to the temperature decay. The parameter *σ* describes the combined broadening effect of the laser pulse and the experimental resolution as limited by the detector. The resulting fit parameters are listed in Table I, and they result in a maximum temperature jump of ΔT of 109 K. Both the diffracted intensity, from one pseudo-Voigt component fit and the width of the peak pumped component (see Fig. 3) remain constant as a function of time.

**FIG. 3.**
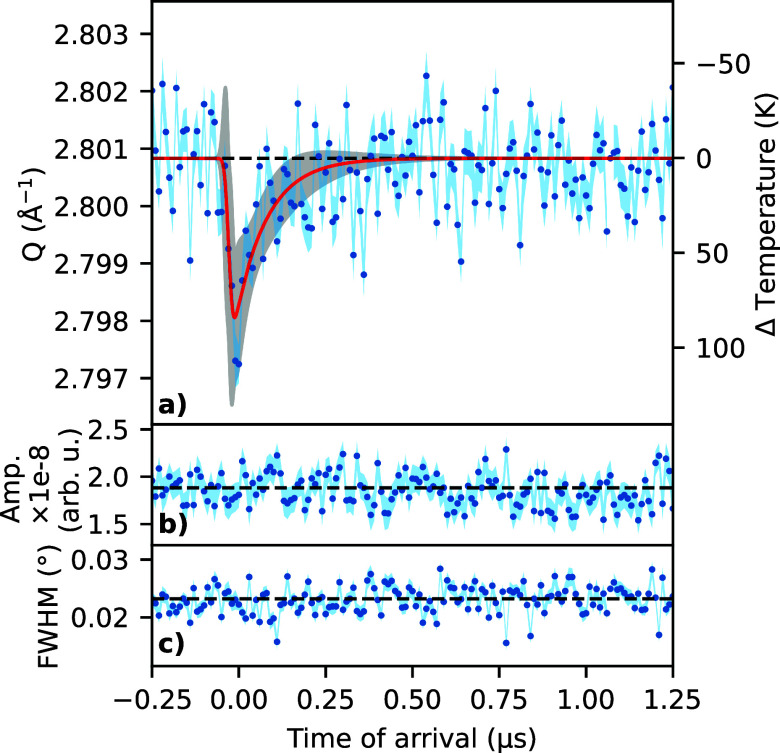
Pd-111 reflection as a function of time of arrival. (a) Fitted momentum transfer, *Q*, of the pumped nanoparticles (blue dots) vs time. The standard error resulting from the peak fitting is indicated as a blue shading. The results from fitting equation (1) to the data are shown as a red line, with the gray shading indicating a 3*σ* uncertainty. Right axis shows the change in temperature as determined from the change in *Q*. (b) Amplitude of the fitted amplitude of peak fitted with one pseudo-Voigt component. (c) The pseudo-Voigt FWHM of the pumped component of the two pseudo-Voigt component fit of the Pd-111 reflections.

**TABLE I.** Values of fitted parameters to the measured data using Eq. (1).

Measurement	Units	*A*	*t*_0_ (ns)	*τ* (ns)	*σ* (ns)
Pd-111, *Q*	Å^–1^	(−3.2 ± 0.4) × 10^–4^	−29 ± 4	91 ± 35	9 ± 6
MgO-111, intensity	counts/10 ns bin	(9.2 ± 0.2) × 10^–5^	−8 ± 1	350 ± 11	15 ± 2

The temporal response of the MgO-111 peak intensity is shown in Fig. 4. There was an insignificant shift in the 2θ diffraction angle of the MgO-111 peak position. The laser's wavelength of 1064 nm (1.17 eV) is significantly below the bandgap of MgO (7.8 eV),^21^ and therefore, it is expected that it interacts only weakly with the substrate. Most likely, the largest contribution to any transient heating of the substrate is coming from the Pd nanoparticles. In addition, the x-ray beam penetrates approximately 100 *μ*m into the substrate, thereby averaging out the effect on the Bragg peak of any thermal-induced lattice expansion decaying with depth into the substrate. The pronounced increase in the MgO peak's integrated intensity was not expected, since diffracted x-ray intensities decrease with increasing temperature due to the Debye–Waller factor. It is known that diffracted beams from MgO single crystals suffer from primary extinction.^22,23^ A laser-induced lattice distortion will deteriorate the crystal's perfection, rendering less extinction effects and a higher diffracted intensity.^24^ The extinction length for the MgO-111 reflex measured with Cu K*α* radiation has been evaluated to be about 5 *μ*m.^25^ Comparison between calculated dynamical reflectivities and those based on kinematic theory shows that extinction can account for several tens of percent intensity loss. Such values have also been reported for weak (hkl odd) MgO reflections.^22^ Based on these considerations, the observation that the 111 reflex's intensity increases by about 20%, which would be in line with the crystal diffraction shifting from dynamical to a more kinematical regime upon laser excitation. Even though the temperature within the x-ray illuminated volume of the MgO does not change, it may well be that a strain wave travels through the crystal causing the lattice distortion, thereby rendering a less perfect crystal. We rule out that the intensity increase is caused by the rigid rotation in combination with the inhomogeneous beam structure (see Fig. 2). The MgO-111 rocking curve shows a single smooth peak with a FWHM of ∼0.01 Å^−1^. The sample was also aligned to the maximum of that curve prior to laser excitation. A shift in the Bragg condition, for example, by thermal expansion, will only lead to a decrease in intensity as the detector in reciprocal space no longer intersect the Bragg peak at its maximum. Additionally, a shift in the MgO-111 peak position due to rotation of the crystal, on the order of ∼0.01 Å^−1^, which may lead to an increase in diffracted intensity, was not observed.

**FIG. 4.**
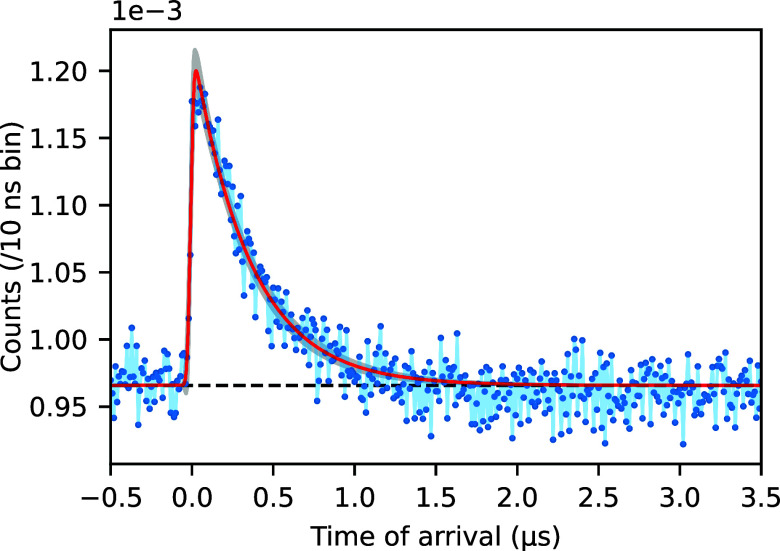
Response of the integrated intensity of the MgO-111 reflection as a function of time fitted exponentially modified Gaussian profile (red). Gray shading represents 3*σ* uncertainty of the fitted profile.

The transient heating of the Pd nanoparticles can be compared with the formalism from a study on a single crystal of platinum using the same nanosecond-laser.^7^ In fact, due to the similarity between the materials properties of Pt and Pd, an almost identical transient heating is expected for the two materials. It is possible the expansion in the lateral and perpendicular direction would differ in the Pd nanoparticles. However, we assume using the formulation for a bulk crystal is a good approximation as we assume surface anchoring of the nanoparticles leading to an asymmetric strain field is minimal. Although the Pd nanoparticles grow epitaxially on the MgO(100) substrate, they are not pseudomorphic. The determined average Pd lattice parameter is the same as the bulk. Even though there is a large lattice mismatch of 7.3%, it has been previously observed that the epitaxial relationship between Pd and MgO involves a coincidence-site-lattice of the first layers of Pd atoms and the MgO(100).^6^ This effect reduces the strain, as would arise from expanding the Pd lattice to match that of MgO. Considering these aspects, it is assumed free lattice expansion of the nanoparticles like that of a single crystal was observed. Using the laser parameters from the present study[App app1], the calculated maximum temperature reached in the case of a Pd bulk single crystal would be $\Delta T_{\text{max}}∼60$ K, which is about 50 K lower than obtained here for the nanoparticles. This discrepancy can be explained by the fact that the MgO support, through which the heat needs to be transported, has a lower thermal conductivity (Appendix). This effect also plays a role for the decay of the Pd nanoparticles heat dissipation, which is fitted to 91 ns, and which is slower than the approximately 50 ns in the case of the platinum single crystal.^7^ The nanoparticles remain hotter for a longer period of time than the near-surface region of a metal. The significantly slower decay of the MgO Bragg peak intensity of 350 ns could be related to a strain wave traveling at the speed of sound (Appendix) through the crystal. Although the details of such a strain wave are unknown at the moment, the timescale matches with a strain wave traversing about 2 mm of MgO, which is twice the crystal thickness.

In conclusion, time-resolved x-ray diffraction experiments were performed determining the laser-induced transient heating of supported Pd nanoparticles. The maximum temperature reached by the Pd nanoparticles is higher and its decay is slower than that expected for a bulk metal crystal. This can be explained by the oxide support's lower heat conductivity, which hampers the dissipation process. The experimental setup consists of a continuous x-ray source in combination with a Timepix3 detector, enabling time-of-arrival photon detection. This type of scheme lowers the total data collection time significantly because there is no need to perform measurements at different pump-probe delay times. The improved generation of these detectors, the Timepix4, will enable faster time resolutions and higher count rates.^26^ It is envisaged that these event based detectors can also be used for time-resolved experiments at synchrotrons and x-ray free electron lasers with a key advantage of efficiently using the limited experiment time available at such facilities.

## Data Availability

The data that support the findings of this study are available from the corresponding author upon reasonable request.

## APPENDIX: MATERIAL PROPERTIES

A list of the material properties of Pd and MgO used in the analysis (Table II).

**TABLE II.** Parameters used in the analysis.

Parameter	Value	Unit
Skin depth (laser) Pd	10	nm
MgO absorption coefficient Cu K*α*	92	cm^−1^
Pd thermal expansion^19^	11.8 × 10^−6^	K^−1^
MgO thermal expansion	10.8 × 10^−6^	K^−1^
MgO heat conductivity^27^	30	WK^−1^ m^−1^
MgO speed of sound^28^	6–8	km s^−1^
Pd heat conductivity^29^	72	WK^−1^ m^−1^
Pd heat capacity^30^	227	JK^−1^ kg^−1^
Pd mass density	12	g cm^−3^
Reflectance 1064 nm	0.8	⋯

## References

[1] D. Cahill , W. Ford , K. Goodson , G. Mahan , A. Majumdar , H. Maris , R. Merlin , and S. Phillpot , J. Appl. Phys. 93, 793 (2003). 10.1063/1.1524305

[2] J.-K. Yu , S. Mitrovic , D. Tham , J. Varghese , and J. R. Heath , Nat. Nanotechnol. 5, 718 (2010). 10.1038/nnano.2010.149 20657598

[3] M. N. Luckyanova , J. Garg , K. Esfarjani , A. Jandl , M. T. Bulsara , A. J. Schmidt , A. J. Minnich , S. Chen , M. S. Dresselhaus , Z. Ren , E. A. Fitzgerald , and G. Chen , Science 338, 936 (2012). 10.1126/science.1225549 23161996

[4] A. Plech , B. Krause , T. Baumbach , M. Zakharova , S. Eon , C. Girmen , G. Buth , and H. Bracht , Nanomaterials 9, 501 (2019). 10.3390/nano9040501 30939755 PMC6523543

[5] P. Nolte , A. Stierle , N. Kasper , N. Y. Jin-Phillipp , N. Jeutter , and H. Dosch , Nano Lett. 11, 4697 (2011). 10.1021/nl2023564 21995433

[6] S. Chung , J.-C. Schober , S. Tober , D. Schmidt , A. Khadiev , D. Novikov , V. Vonk , and A. Stierle , ACS Nano 15, 13267 (2021). 10.1021/acsnano.1c03002 34350766

[7] R. Shayduk , V. Vonk , B. Arndt , D. Franz , J. Strempfer , S. Francoual , T. F. Keller , T. Spitzbart , and A. Stierle , Appl. Phys. Lett. 109, 043107 (2016). 10.1063/1.4959252

[8] K.-D. Liss , A. Magerl , R. Hock , B. Waibel , and A. Remhof , in *Time Structure of X-Ray Sources and Its Applications*, edited by A. K. Freund , H. P. Freund , and M. R. Howells ( SPIE Press, 1998), Vol. 3451, pp. 117–127.

[9] T. Poikela , J. Plosila , T. Westerlund , M. Campbell , M. D. Gaspari , X. Llopart , V. Gromov , R. Kluit , M. V. Beuzekom , F. Zappon , V. Zivkovic , C. Brezina , K. Desch , Y. Fu , and A. Kruth , J. Instrum. 9, C05013 (2014). 10.1088/1748-0221/9/05/C05013

[10] X. Llopart , R. Ballabriga , M. Campbell , L. Tlustos , and W. Wong , Nucl. Instrum. Methods Phys. Res. Sect. A 581, 485 (2007). 10.1016/j.nima.2007.08.079

[11] E. Frojdh , M. Campbell , M. De Gaspari , S. Kulis , X. Llopart , T. Poikela , and L. Tlustos , J. Instrum. 10, C01039 (2015). 10.1088/1748-0221/10/01/C01039

[12] A. Stierle , T. F. Keller , H. Noei , V. Vonk , and R. Roehlsberger , J. Large-Scale Res. Facilities 2, A76–A76 (2016). 10.17815/jlsrf-2-140

[13] L. G. Parratt , Phys. Rev. 95, 359 (1954). 10.1103/PhysRev.95.359

[14] A. Al-Refaie , M. Johny , J. Correa , D. Pennicard , P. Svihra , A. Nomerotski , S. Trippel , and J. Küpper , J. Instrum. 14, P10003 (2019). 10.1088/1748-0221/14/10/P10003

[15] M. Lohmeier and E. Vlieg , J. Appl. Crystallogr. 26, 706 (1993). 10.1107/S0021889893004868

[16] F. Pitters , N. Alipour Tehrani , D. Dannheim , A. Fiergolski , D. Hynds , W. Klempt , X. Llopart , M. Munker , A. Nürnberg , S. Spannagel , and M. Williams , arXiv:1901.07007 (2019).

[17] S. Tsigaridas , M. V. Beuzekom , H. V. Graaf , F. Hartjes , K. Heijhoff , N. P. Hessey , P. J. de Jong , and V. Prodanovic , Nucl. Instrum. Methods Phys. Res. Sect. A 930, 185 (2019). 10.1016/j.nima.2019.03.077

[18] H. Yousef , G. Crevatin , E. N. Gimenez , I. Horswell , D. Omar , and N. Tartoni , Nucl. Instrum. Methods Phys. Res. Sect. A 845, 639 (2017). 10.1016/j.nima.2016.04.075

[19] Y. Kuru , M. Wohlschloegel , U. Welzel , and E. J. Mittemeijer , Surf. Coat. Technol. 202, 2306 (2008). 10.1016/j.surfcoat.2007.08.002

[20] E. Grushka , Anal. Chem. 44, 1733 (1972). 10.1021/ac60319a011 22324584

[21] R. Whited , C. J. Flaten , and W. Walker , Solid State Commun. 13, 1903 (1973). 10.1016/0038-1098(73)90754-0

[22] J. L. Lawrence , Acta Crystallogr. Sect. A 29, 208 (1973). 10.1107/S0567739473000495

[23] V. G. Tsirelson , A. S. Avilov , Y. A. Abramov , E. L. Belokoneva , R. Kitaneh , and D. Feil , Acta Crystallogr. Sect. B 54, 8 (1998). 10.1107/S0108768197008963

[24] K. Liss , A. Magerl , A. Remhof , and R. Hock , Acta Crystallogr. Sect. A 53, 181 (1997). 10.1107/S010876739601327X

[25] B. E. Warren , *X-Ray Diffraction* ( Dover Publications, Inc., New York, 1990).

[26] X. Llopart , J. Alozy , R. Ballabriga , M. Campbell , R. Casanova , V. Gromov , E. Heijne , T. Poikela , E. Santin , V. Sriskaran , L. Tlustos , and A. Vitkovskiy , J. Instrum. 17, C01044 (2022). 10.1088/1748-0221/17/01/C01044

[27] A. Slifka , B. Filla , and J. Phelps , J. Res. Nat. Inst. Standards Technol. 103, 357 (1998). 10.6028/jres.103.021 PMC4887202 28009383

[28] A. Chopelas , Earth Planet. Sci. Lett. 114, 185 (1992). 10.1016/0012-821X(92)90160-W

[29] C. Y. Ho , R. W. Powell , and P. E. Liley , J. Phys. Chem. Ref. Data 1, 279 (2009). 10.1063/1.3253100

[30] G. T. Furukawa , M. L. Reilly , and J. S. Gallagher , J. Phys. Chem. Ref. Data 3, 163 (2009). 10.1063/1.3253137

